# The invasion of de-differentiating cancer cells into hierarchical tissues

**DOI:** 10.1371/journal.pcbi.1007167

**Published:** 2019-07-01

**Authors:** Da Zhou, Yue Luo, David Dingli, Arne Traulsen

**Affiliations:** 1 School of Mathematical Sciences and Fujian Provincial Key Laboratory of Mathematical Modeling and High-Performance Scientific Computation, Xiamen University, Xiamen, People’s Republic of China; 2 Department of Evolutionary Theory, Max Planck Institute for Evolutionary Biology, Plön, Germany; 3 Division of Hematology and Department of Internal Medicine, Mayo Clinic, Rochester, Minnesota, United States of America; University of California Irvine, UNITED STATES

## Abstract

Many fast renewing tissues are characterized by a hierarchical cellular architecture, with tissue specific stem cells at the root of the cellular hierarchy, differentiating into a whole range of specialized cells. There is increasing evidence that tumors are structured in a very similar way, mirroring the hierarchical structure of the host tissue. In some tissues, differentiated cells can also revert to the stem cell phenotype, which increases the risk that mutant cells lead to long lasting clones in the tissue. However, it is unclear under which circumstances de-differentiating cells will invade a tissue. To address this, we developed mathematical models to investigate how de-differentiation is selected as an adaptive mechanism in the context of cellular hierarchies. We derive thresholds for which de-differentiation is expected to emerge, and it is shown that the selection of de-differentiation is a result of the combination of the properties of cellular hierarchy and de-differentiation patterns. Our results suggest that de-differentiation is most likely to be favored provided stem cells having the largest effective self-renewal rate. Moreover, jumpwise de-differentiation provides a wider range of favorable conditions than stepwise de-differentiation. Finally, the effect of de-differentiation on the redistribution of self-renewal and differentiation probabilities also greatly influences the selection for de-differentiation.

## Author summary

How can a tissue such as the blood system or the skin, which constantly produces a huge number of cells, avoids that errors accumulate in the cells over time? Such tissues are typically organized in cellular hierarchies, which induce a directional relation between different stages of cellular differentiation, minimizing the risk of retention of mutations. However, recent evidence also shows that some differentiated cells can de-differentiate into the stem cell phenotype. Why does de-differentiation arise in some tumors, but not in others? We developed a mathematical model to study the growth competition between de-differentiating mutant cell populations and non de-differentiating resident cell population. Our results suggest that the invasion of de-differentiation is jointly influenced by the cellular hierarchy (e.g. number of cell compartments, inherent cell division pattern) and the de-differentiation pattern, i.e. how exactly cells acquire their stem-cell like properties.

## Introduction

**Fig 1:**
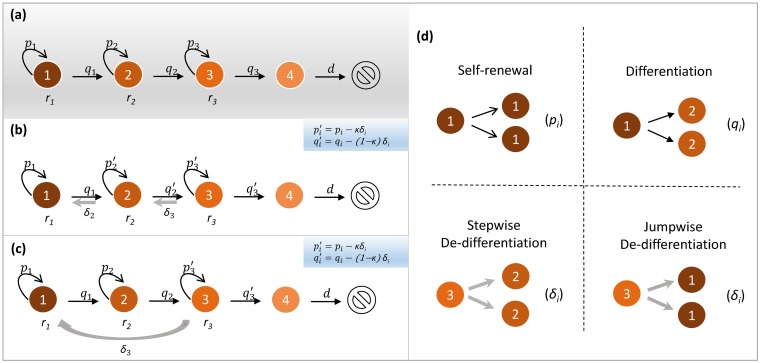
Representation of our models. We illustrate our models by considering a four-compartment hierarchical structure. (**a**) Null model without de-differentiation. Each compartment represents a certain stage of cell differentiation. For example, compartment 1 represents stem cell which performs cell division with rate *r*_1_. In each cell division, it can either give birth to two identical stem cells (self-renewal) with probability *p*_1_ or two identical daughter cells in adjacent downstream compartment 2 (differentiation) with probability *q*_1_. Similar division pattern can also happen to cells in compartments 2 and 3 (with division rates *r*_2_ and *r*_3_ respectively). Compartment 4 represents terminally differentiated cells which cannot divide and are removed from the tissue at rate *d*. (**b**) Stepwise de-differentiation. Based on the hierarchical structure, we consider de-differentiation from downstream compartment *i* + 1 to the adjacent upstream compartment *i*. By introducing de-differentiation (with probability *δ*_*i*_) in cell division, the self-renewal probability of each cell in compartment *i* is changed from *p*_*i*_ to *p*_*i*_ − *κδ*_*i*_, while its differentiation probability is changed from *q*_*i*_ to *q*_*i*_ − (1 − *κ*)*δ*_*i*_. Here, we have introduced the redistributing factor *κ* that captures the effect of de-differentiation on the self-renewal and differentiation probabilities. (**c**) Jumpwise de-differentiation, in which de-differentiation happens directly from compartment 3 to 1 without cells reaching the state in compartment 2. For each cell in compartment 3, its self-renewal probability is changed from *p*_3_ to *p*_3_ − *κδ*_3_, and its differentiation probability is changed from *q*_3_ to *q*_3_ − (1 − *κ*)*δ*_3_. (**d**) The four cell division patterns used in our models.

In multicellular organisms, it is important that the inevitable replication errors of cells do not persist and threaten the functioning of the organism as a whole. Many tissues that need to undergo continuous cell turnover are organized in a hierarchical multi-compartment structure, which reduces the risk of the persistence of such mutations [1–13]. Each compartment represents a certain stage of cellular differentiation (Fig 1). At the root of the cellular hierarchy are tissue specific stem cells (SCs), which are capable of self-renewal and differentiation into more mature cells [14]. It is often argued that cancers may have similar hierarchical structures, where cancer stem cells (CSCs) possess characteristics associated with SCs in normal tissues [14, 15]. The CSCs scenario assumes that some cancerous tissues are hierarchically organized, similar to normal tissues [16].

The hierarchical tissue architecture proposes a unidirectional cascade from less differentiated stages to more differentiated stages (Fig 1a). This would minimize the risk of the accumulation of genomic damage in the long-term self-renewing stem cells. However, there is significant evidence that the directional relation between different stages of differentiation could be broken in some tissues [17–22]. In other words, cells in later differentiated stages can, under some circumstances, revert to earlier differentiated stages, or even the stem cell stage, in a process called de-differentiation (Fig 1b and 1c). De-differentiation could play an important role in regeneration and tumorigenesis [17]. In particular, even though the origin of cancer stem cells is still an open question, growing evidence shows that non-stem cancer cells can reacquire stem-like characteristics in colon cancer [23], breast cancer [20, 21], melanoma [24], leukemia [25–28], glioblastoma [29], and other cancers. For example, expression of the MLL-AF9 gene in committed hematopoietic progenitor cells led to the development of a leukemic stem cell population where only four of these cells were able to result in disease in a mouse model that could be transferred from one mouse to another, confirming the presence of a stem cell population [27].

More recently, special attention has been paid to the effect of de-differentiation on the cellular hierarchy by mathematically modeling its impact [30]. Previous work has e.g. considered how de-differentiation influences the waiting time to carcinogenesis [31], the fixation probability of a mutant [32, 33], the phenotypic equilibrium [34–36], transient overshoots [37, 38], and radiation sensitivity [29]. However, the adaptive significance of de-differentiation is still poorly understood: Under which circumstances would de-differentiation arise in the first place and rise in abundance? Intuitively, de-differentiation contributes to a faster growth of stem cells, and note that stem cells are typically defined as having the greatest self-renewal potential, hence de-differentiation should benefit the growth of whole population and always be favored in the cellular hierarchy. However, reality seems even more complicated, as de-differentiation arises in only some tumors, but not in others. Therefore, it is still unclear whether de-differentiation is a crucial improvement or just an unintended consequence of cellular hierarchy. Moreover, the comparison between different patterns of de-differentiation has received little attention.

Here, we develop a matrix population model [39] of a stage-structured population for studying the evolution of de-differentiation. Two typical de-differentiation cases are taken into account in our model: One is stepwise de-differentiation which happens from a downstream compartment to an adjacent upstream compartment (Fig 1b), the other is jumpwise de-differentiation which is directly from a highly differentiated compartment into the stem cell compartment without any intermediate stages (Fig 1c). Given a hierarchically structured multi-compartment cell population, we are concerned about the selection of stepwise or jumpwise de-differentiating mutant cell population in the competition with non de-differentiating resident cell population. By comparing the growth rates of different cell populations, we analyze favorable conditions for different de-differentiation patterns to invade a tissue. However, we do not study the direct competition between de-differentiating and non de-differentiating cells. We hope that our work contributes to the theoretical understanding of the emergence of de-differentiation in multicellular tissues.

## Methods

### The matrix population model for cellular hierarchy

Consider a cell population composed of *n* compartments, each of which represents a certain stage of differentiation [10, 13] (Fig 1). For example, compartment 1 represents stem cells, and compartment *n* represents terminally differentiated cells. Each cell in compartment *i* (1 ≤ *i* ≤ *n* − 1) divides at rate *r*_*i*_. With probability *p*_*i*_, it divides symmetrically, giving birth to two identical cells in compartment *i* (Fig 1d). With probability *q*_*i*_, it differentiates symmetrically, generating two identical daughter cells in compartment *i* + 1. The terminally differentiated cells in compartment *n* cannot divide and are removed from the tissue at rate *d*.

We use the vector $\vec{N}=(N_{1},N_{2},...,N_{n}{)}^{T}$ to denote the cell numbers in different compartments. Then, the hierarchically structured population dynamics composed of non de-differentiating cells can be described as a matrix population model [39]

$$\frac{d\vec{N}}{dt}=A_{0}\vec{N},$$ (1)

where *A*_0_ is the projection matrix which is given by

$$\begin{array}{c} A_{0}=(\begin{array}{ccccc} r_{1}\left(p_{1}-q_{1}\right) & 0 & \cdots & \cdots & 0\\ 2r_{1}q_{1} & r_{2}\left(p_{2}-q_{2}\right) & \cdots & \cdots & 0\\ 0 & 2r_{2}q_{2} & \cdots & \cdots & 0\\ 0 & 0 & \cdots & 0 & 0\\ \vdots & \vdots & \ddots & 0 & 0\\ \vdots & \vdots & \cdots & r_{n-1}\left(p_{n-1}-q_{n-1}\right) & 0\\ 0 & 0 & \cdots & 2r_{n-1}q_{n-1} & -d \end{array}). \end{array}$$ (2)

Here *r*_*i*_(*p*_*i*_ − *q*_*i*_) represents the effective self-renewal rate of compartment *i*, and 2*r*_*i*_*q*_*i*_ represents the influx rate from compartment *i* to compartment *i* + 1 due to differentiation. It should be pointed out that, for simplicity, asymmetric division [40, 41] (giving birth to one daughter cell in compartment *i* and the other in compartment *i* + 1) is not taken into account here. It can be shown that our model is equivalent to a model with asymmetric division [42]. Actually, by introducing asymmetric division (e.g. with probability *s*_*i*_) into our model, the effective self-renewal rate of compartment *i* is still given by *r*_*i*_(*p*_*i*_ − *q*_*i*_), while the influx rate from compartment *i* to compartment *i* + 1 is shifted from 2*r*_*i*_*q*_*i*_ to 2*r*_*i*_*q*_*i*_ + *r*_*i*_*s*_*i*_. We can see that the characteristics of matrix *A*_0_, such as essentially non-negativity (all the off-diagonal elements are non-negative [43]) and lower triangular structure, remain unchanged. Therefore, our approaches and results are still applicable for the model with asymmetric division.

Let $M\left(t\right)=\sum_{i=1}^{n}N_{i}\left(t\right)$ be the total cell number of the population. Note that *A*_0_ is an essentially non-negative and lower triangular matrix. According to the standard theory of matrix population models [39], the population approaches exponential growth, i.e.

$$M\left(t\right)\approx M\left(0\right)\text{exp}\left[\lambda_{0}t\right]\;\;\text{for}\;\text{large}\;t,$$ (3)

where λ_0_ is the real largest eigenvalue. The largest eigenvalue hence characterizes the asymptotic growth rate of the whole population, which is often used as a measure of fitness in matrix population models [44, 45]. The whole population will expand if λ_0_ > 0, remain in homeostasis if λ_0_ = 0, or shrink if λ_0_ < 0. Here, we are interested in the cases when λ_0_ ≥ 0, i.e. we assess whether a mutant can invade an expanding or steady resident population by comparing their fitness measures. Besides, due to the intense inevitable internal and external noise in cellular dynamics [46] and experimental measurements, in reality it is quite unlikely for different compartments to have exactly the same observations of parameters, and therefore there is little chance for *A*_0_ to have multiple eigenvalues [37]. It is thus reasonable to assume that λ_0_ is unique (or simple).

### Stepwise and jumpwise de-differentiation

Let us now introduce de-differentiation processes given the non de-differentiating resident cell population Eq (1). Since it is biologically unclear how a non de-differentiating resident cell acquires the ability for de-differentiation, here we consider de-differentiation as a result of certain genetic or epigenetic alterations (jointly referred to as mutations). It is assumed that the mutant cells are provided with the additional ability of de-differentiation. More specifically, when these mutant cells divide, besides symmetric division and symmetric differentiation, they can also perform symmetric de-differentiation (Fig 1d) with a small probability. In principle, there are two different ways to do this: (i) stepwise de-differentiation, where cells de-differentiate to the previous compartment, and (ii) jumpwise de-differentiation, where de-differentiation happens across multiple compartments at a time. These are the most extreme cases and a mixture between them is possible.

For stepwise de-differentiation, a mutant cell in compartment *i* gives rise to two daughter cells in its adjacent upstream compartment *i* − 1 (Fig 1b) when de-differentiation happens. Suppose that the de-differentiation probability from compartment *i* to *i* − 1 is *δ*_*i*_. Then, the influx rate from compartment *i* to *i* − 1 due to de-differentiation is given by 2*r*_*i*_ *δ*_*i*_. We denote the self-renewal and differentiation probabilities of each mutant cell in compartment *i* as $p_{i}^{\prime }$ and $q_{i}^{\prime }$ respectively. Note that $p_{i}^{\prime }+q_{i}^{\prime }+\delta_{i}=1$, that is, the sum of the self-renewal and differentiation probabilities of each mutant cell is reduced from 1 to 1 − *δ*_*i*_. Due to the current lack of knowledge regarding the effect of de-differentiation on the self-renewal and differentiation probabilities, there is no way to know how much the self-renewal probability or differentiation probability changes individually. In view of this, we introduce a parameter *κ* (0 ≤ *κ* ≤ 1) to capture how mutant cell redistributes the probabilities for self-renewal and differentiation when taking de-differentiation into account. We thus call *κ* the redistributing factor. In this way, the self-renewal probability of each mutant cell in compartment *i* is given by $p_{i}^{\prime }=p_{i}-\kappa \delta_{i}$, and its differentiation probability is given by $q_{i}^{\prime }=q_{i}-\left(1-\kappa \right)\delta_{i}$. Although currently we are unable to measure the specific value of *κ*, it would be very interesting to see if the redistributing factor affects the emergence of de-differentiation, and we will see that *κ* does deserve special attention.

It has been reported that de-differentiation is generally a rare event [21], we thus assume that *ρ*_*i*_ = 2*r*_*i*_*δ*_*i*_ ≪ 1. As the occurrence of de-differentiation for different stages of differentiation is poorly understood, for simplicity we assume that all the *ρ*_*i*_ are the same, i.e. they are independent of index *i* and denoted as *ρ* for short. In this way, the population dynamics of the stepwise de-differentiating mutant cell population can be modeled with a projection matrix given by

$$\begin{array}{c} A_{S}=(\begin{array}{cccccc} r_{1}\left(p_{1}-q_{1}\right) & \rho & \cdots & \cdots & \cdots & 0\\ 2r_{1}q_{1} & r_{2}\left(p_{2}-q_{2}\right)-\kappa \rho & \cdots & \cdots & \cdots & 0\\ 0 & 2r_{2}q_{2}-\left(1-\kappa \right)\rho & \cdots & \cdots & \cdots & 0\\ 0 & 0 & \ddots & \vdots & 0 & 0\\ \vdots & \vdots & \vdots & \ddots & \rho & 0\\ \vdots & \vdots & \cdots & \cdots & r_{n-1}\left(p_{n-1}-q_{n-1}\right)-\kappa \rho & 0\\ 0 & 0 & \cdots & \cdots & 2r_{n-1}q_{n-1}-\left(1-\kappa \right)\rho & -d \end{array}). \end{array}$$ (4)

Jumpwise de-differentiation provides an alternative pattern where even highly differentiated cells can directly revert to stem cells without being in intermediate stages (Fig 1c). Formally, it is assumed that the jumpwise de-differentiating mutant cell in compartment *n* − 1 can give birth to two daughter stem cells in compartment 1 (Fig 1d). Therefore, the projection matrix is given by

$$\begin{array}{c} A_{J}=(\begin{array}{cccccc} r_{1}\left(p_{1}-q_{1}\right) & 0 & 0 & \cdots & \rho & 0\\ 2r_{1}q_{1} & r_{2}\left(p_{2}-q_{2}\right) & 0 & \cdots & \cdots & 0\\ 0 & 2r_{2}q_{2} & r_{3}\left(p_{3}-q_{3}\right) & \cdots & \cdots & 0\\ 0 & 0 & \ddots & \cdots & 0 & 0\\ \vdots & \vdots & \vdots & \ddots & 0 & 0\\ \vdots & \vdots & \cdots & \cdots & r_{n-1}\left(p_{n-1}-q_{n-1}\right)-\kappa \rho & 0\\ 0 & 0 & \cdots & \cdots & 2r_{n-1}q_{n-1}-\left(1-\kappa \right)\rho & -d \end{array}). \end{array}$$ (5)

### Selection gradient for de-differentiation

In the following, we consider the competition between a non de-differentiating resident cell population and a stepwise de-differentiating mutant cell population (which is called *S* mutant cell population for short), as well as between a non de-differentiating resident cell population and a jumpwise de-differentiating mutant cell population (which is called *J* mutant cell population for short) by comparing their fitness measures, i.e. the largest eigenvalues λ_0_, λ_*S*_ and λ_*J*_ of *A*_0_, *A*_*S*_ and *A*_*J*_, respectively. Note that *ρ* is very small, such that both *A*_*S*_ and *A*_*J*_ can be seen as matrix perturbations to *A*_0_. According to the eigenvalue perturbation theory (see e.g. Theorem 4.4 in [47]), both λ_*S*_ and λ_*J*_ are differentiable with respect to *ρ* provided that λ_0_ is simple. In this way, we have

$$\lambda_{S}\approx \lambda_{0}+\Delta \lambda_{S}\rho ,\;\;\;\lambda_{J}\approx \lambda_{0}+\Delta \lambda_{J}\rho .$$ (6)

Here, Δλ_*S*_ and Δλ_*J*_ are given by

$$\Delta \lambda_{S}={\vec{\mu }}^{T}[\frac{\partial A_{S}}{\partial \rho }{]}_{\rho =0}\vec{\eta },\;\;\Delta \lambda_{J}={\vec{\mu }}^{T}[\frac{\partial A_{J}}{\partial \rho }{]}_{\rho =0}\vec{\eta },$$ (7)

where $\vec{\mu }$ and $\vec{\eta }$ are the left and right eigenvectors associated with λ_0_ respectively (see S1 File).

For a given parameter set (*r*_*i*_, *p*_*i*_, *q*_*i*_, *d*, *κ*), Δλ_*S*_ characterizes the selective difference between an *S* mutant cell population and a non de-differentiating cell population. If Δλ_*S*_ > 0, for example, the *S* mutant population is favored in this competition—a non de-differentiating resident cell population is invaded by an *S* mutant cell population. Therefore, Δλ_*S*_ corresponds to a selection gradient and acts as a comparative fitness measure of the *S* mutant cell population relative to the non de-differentiating resident cell population. A similar argument also applies for Δλ_*J*_. We thus term Δλ_*S*_ and Δλ_*J*_ as selection gradients of the *S* mutant cell population and the *J* mutant cell population, respectively. Based on these quantities, we will analyze the favorable conditions for de-differentiation.

## Results

We infer whether de-differentiation leads to an increased fitness in the different cases (stepwise and jumpwise), both analytically and numerically.

Let us first focus on the null model without de-differentiation. In this case, the projection matrix *A*_0_ is a lower triangular matrix whose eigenvalues are just the diagonal elements. Note that the resident cell population in Eq (1) is assumed to be not shrinking, which implies that there exists at least one non-negative diagonal element in *A*_0_. In this way, the largest eigenvalue λ_0_ is the largest among all the non-negative diagonal elements of *A*_0_. Note that −*d* is always negative, such that λ_0_ is always in the form of $r_{j_{0}}\left(p_{j_{0}}-q_{j_{0}}\right)$, where *j*_0_ is the compartment that maximizes this quantity.

**Fig 2:**
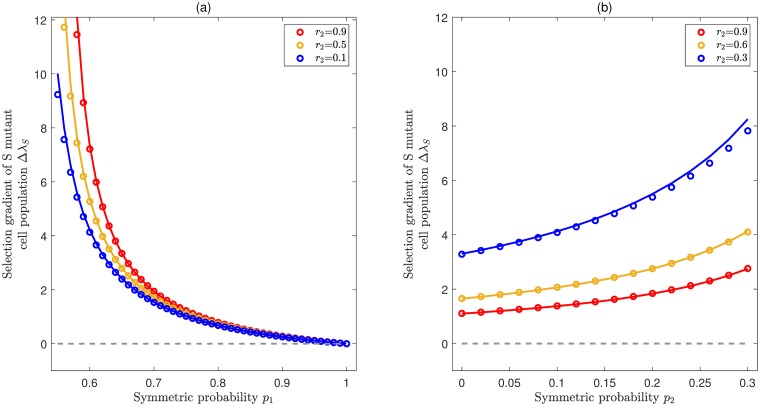
Selection for stepwise de-differentiation when the effective rate of self renewal is highest for stem cells. Illustration of the selection gradient (comparative fitness) of the *S* mutant cell population Δλ_*S*_ as a function of division rates and symmetric division probabilities, provided that the stem cell compartment has the largest effective self-renewal rate, i.e. λ_0_ = *r*_1_(*p*_1_ − *q*_1_). In both panels, colored lines represent analytical approximations from Eq (8) by using the eigenvalue perturbation method and symbols represent exact numerical solutions, which agree very well with each other. The common parameters are *n* = 4, *κ* = 0.1, *ρ* = 0.001, *d* = 0.05, *r*_1_ = 0.99, *r*_3_ = 0.3. (**a**) **Expanding case** (λ_0_ > 0). De-differentiation provides a fitness advantage for all values of *p*_1_ and *r*_2_. Here *p*_2_ = 0.55, *p*_3_ = 0.6 and the range of *p*_1_ (0.55 < *p*_1_ < 1.0) ensures that *r*_1_(*p*_1_ − *q*_1_) is the largest eigenvalue. (**b**) **Homeostasis case** (λ_0_ = 0). De-differentiation also provides a fitness advantage for all values of *p*_2_ and *r*_2_. Here *p*_1_ = 0.5, *p*_3_ = 0 and the range of *p*_2_ (0 < *p*_1_ < 0.3) ensures that λ_0_ = 0 is the largest eigenvalue.

Next, we turn to stepwise de-differentiation, Eq (4). Given $\lambda_{0}=r_{j_{0}}\left(p_{j_{0}}-q_{j_{0}}\right)$, the selection gradient (comparative fitness) of an *S* mutant cell population is given by (see S1 File for mathematical details)

$$\begin{array}{c} \Delta \lambda_{S}=\{\begin{array}{cc} \Gamma_{1,1,2}\; & \text{for}\;j_{0}=1\\ \Gamma_{j_{0}-1,j_{0},j_{0}-1}+\Gamma_{j_{0},j_{0},j_{0}+1}-\kappa \; & \text{for}\;1<j_{0}<n-1\\ \Gamma_{n-2,n-1,n-2}-\kappa \; & \text{for}\;j_{0}=n-1 \end{array} \end{array}$$ (8)

where $\Gamma_{j,k,l}=\frac{2r_{j}q_{j}}{r_{k}\left(p_{k}-q_{k}\right)-r_{l}\left(p_{l}-q_{l}\right)}$. Note that the largest eigenvalue λ_0_ is unique, which implies that $r_{j_{0}}\left(p_{j_{0}}-q_{j_{0}}\right)$ is strictly larger than any other *r*_*j*_(*p*_*j*_ − *q*_*j*_) for *j* ≠ *j*_0_. Thus, all the Γ_*j*,*k*,*l*_ in Eq (8) are positive. In particular, for *j*_0_ = 1, Δλ_*S*_ = Γ_1,1,2_ is positive. In other words, an *S* mutant cell population is always favored in the competition with non de-differentiating resident cell population provided that stem cells have the largest effective self-renewal rate among all cell compartments. We performed exact numerical solutions to verify our theoretical approximation and find a very good agreement. Fig 2 illustrates two different cases. One is for expanding populations, i.e. when the effective self-renewal rate of stem cells λ_0_ = *r*_1_(*p*_1_ − *q*_1_) is positive. The other is for the populations at steady state (homeostasis), i.e. when λ_0_ is zero. We can see that the selection gradient Δλ_*S*_ is always positive, even though different patterns of function relation are present for left and right panels. That is, the stepwise de-differentiation always provides a fitness advantage, regardless of whether the resident cell populations are expanding or at steady state. Actually, this result is quite in line with biological intuition. Given that stem cells have the highest self-renewal potential, i.e. the self-renewal potential is gradually lost in the process of differentiation, de-differentiation effectively leads to a faster growth rate of the population.

In general, stem cells are defined as having the greatest potential for long term self-renewal. There is also evidence that stem cells replicate slowly and therefore in many tissues it is the progenitor cells that lead to amplification and maintenance of tissues due to a process of replication, self-renewal and differentiation [48, 49]. Previous modeling work has considered different relationships between differentiation stage and self-renewal rate [10, 50, 51], in which downstream compartments rather than the stem cells compartment were often assumed to have the largest effective self-renewal rate. Therefore, it is of significance and interest to consider the case of *j*_0_ > 1 in our model.

**Fig 3:**
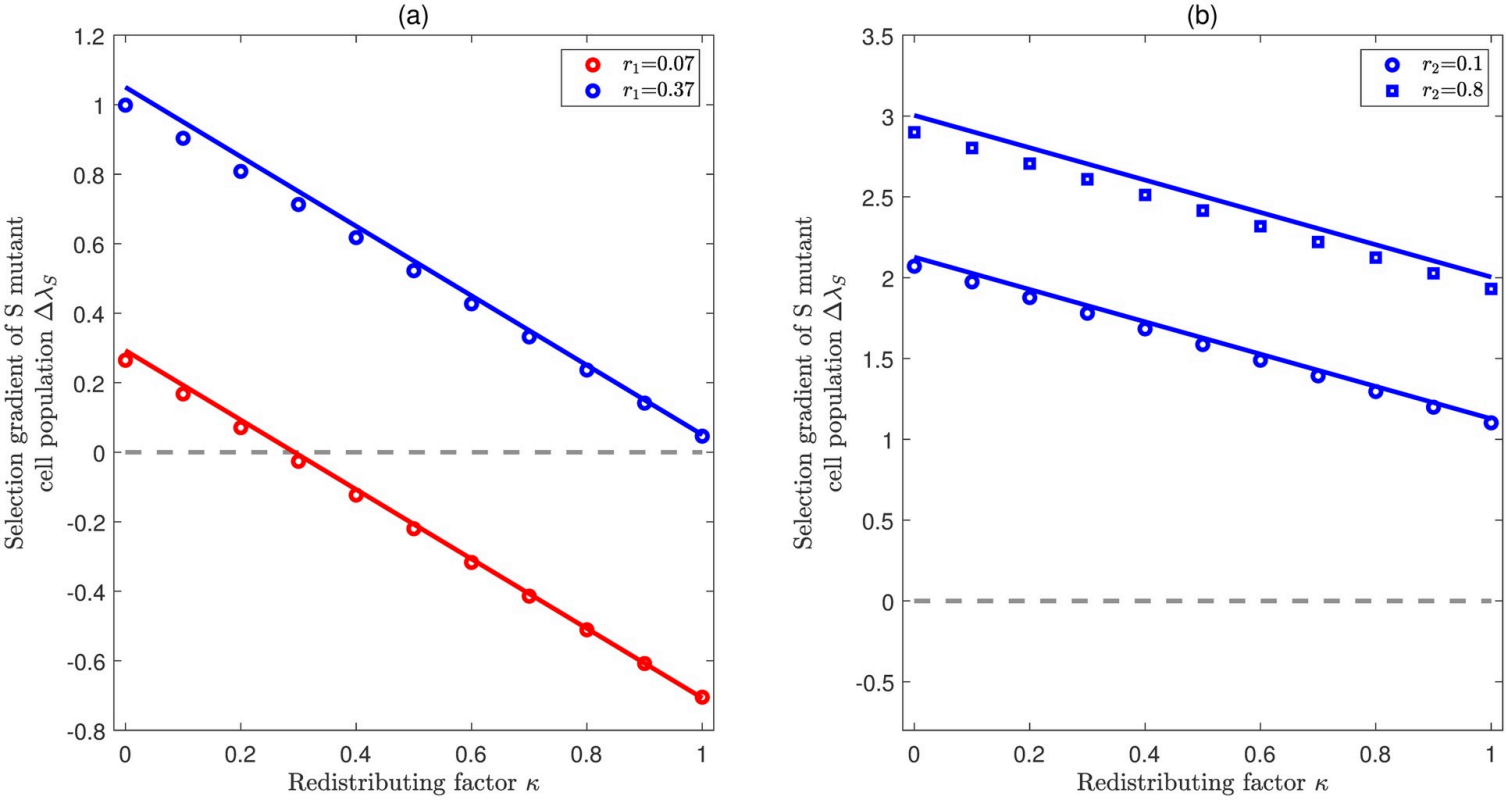
Selection for stepwise de-differentiation when the effective rate of self renewal is highest in compartment 2. Illustration of the selection gradient (comparative fitness) of the *S* mutant cell population Δλ_*S*_ as a function of redistributing factor and division rates provided that λ_0_ = *r*_2_(*p*_2_ − *q*_2_). In both panels, colored lines represent the eigenvalue perturbation results from Eq (8) and symbols represent exact numerical solutions. The common parameters are *n* = 4, *ρ* = 0.01, *d* = 0.05. (**a**) **Expanding case** (λ_0_ > 0). In this case, there are two different scenarios: For $r_{1}<\frac{r_{2}\left(2p_{2}-1\right)\left(\Gamma_{2,2,3}-1\right)}{\left(2p_{1}-1\right)\left(\Gamma_{2,2,3}-1\right)-2\left(1-p_{1}\right)}\approx 0.1950$, Δλ_*S*_ is always positive (blue color); For *r*_1_ > 0.1950, Δλ_*S*_ changes from positive to negative with the increase of *κ* (red color). Here *p*_1_ = 0.5, *p*_2_ = 0.95, *p*_3_ = 0.55, *r*_2_ = 0.44, and *r*_3_ = 0.17. (**b**) **Homeostasis case** (λ_0_ = 0). In this case, Δλ_*S*_ is always positive. Here *p*_1_ = 0.001, *p*_2_ = 0.5, *p*_3_ = 0.001, *r*_1_ = 0.99, and *r*_3_ = 0.8.

**Fig 4:**
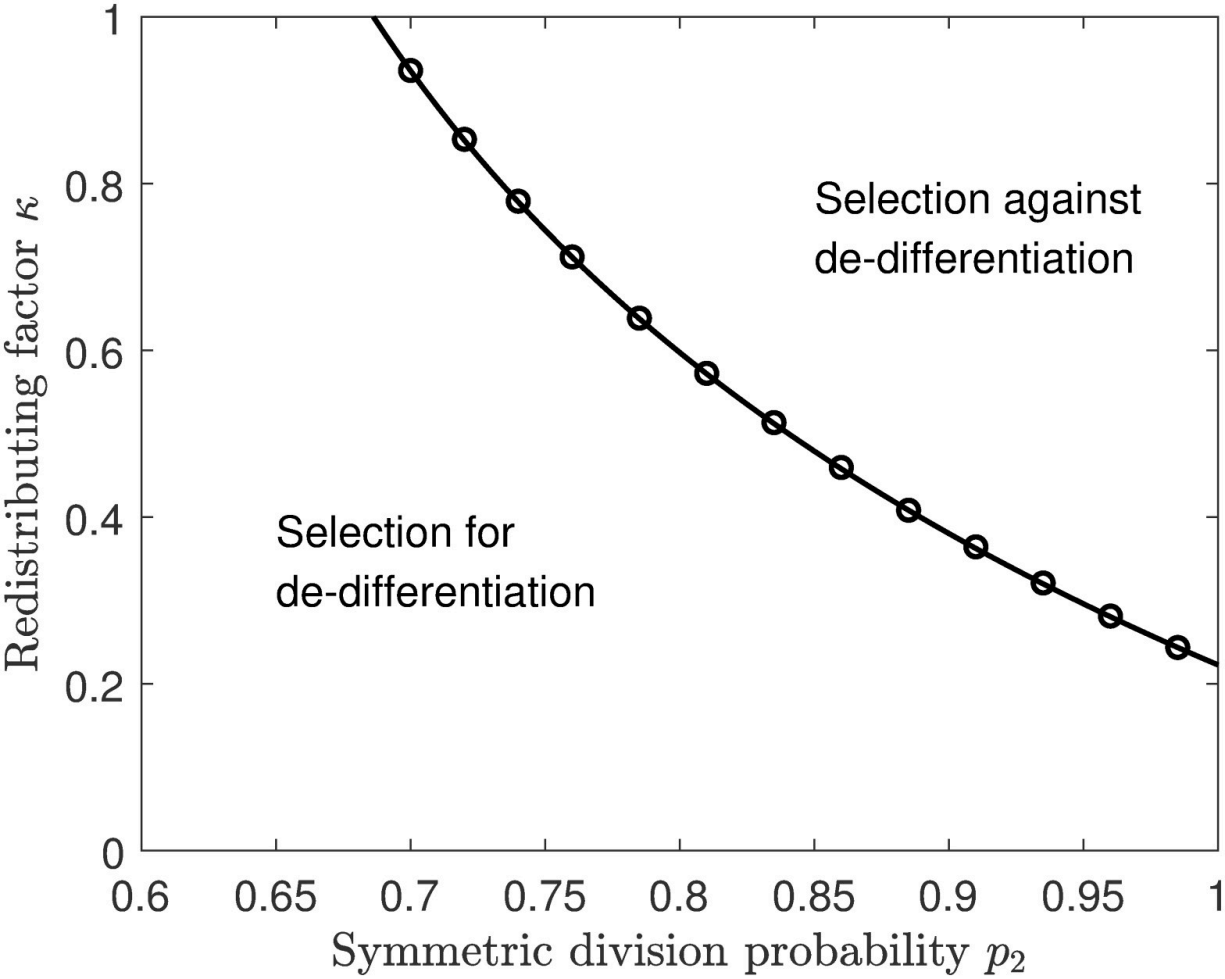
Selection for stepwise de-differentiation in a landscape composed of the symmetric division probability *p*_2_ and redistributing factor *κ* when the effective rate of self renewal is highest in compartment 2. The curve represents the boundary with Δλ_*S*_ = 0, which is generated by the eigenvalue perturbation approximation from Eq (8). The symbols represent the exact numerical solutions for Δλ_*S*_ = 0. The parameters are *n* = 4, *ρ* = 0.01, *r*_1_ = 0.0885, *r*_2_ = 0.4145, *r*_3_ = 0.5555, *p*_1_ = 0.4723, *p*_3_ = 0.0727, *d* = 0.005.

From Eq (8) we can see that Δλ_*S*_ is a linear combination of Γ_*j*,*k*,*l*_ and *κ* when *j*_0_ > 1. It is interesting to see that Δλ_*S*_ is negatively correlated with *κ*. Note that *κ* is the redistributing factor that characterizes how the introduction of de-differentiation reshapes the probabilities for self-renewal and differentiation. For *κ* = 0, Δλ_*S*_ is surely positive. With an increase of *κ*, Δλ_*S*_ could become negative. Hence, there are typically two scenarios of Δλ_*S*_: either it is always larger than zero for any *κ*, or it changes from positive to negative at some critical point 0 < κ* < 1. Fig 3 illustrates how Δλ_*S*_ changes with *κ* provided that compartment 2 has the largest effective self-renewal rate (*j*_0_ = 2). In the expanding case (left panel) both of these two scenarios are present, whereas in the homeostasis case (right panel) Δλ_*S*_ is always larger than zero. Actually, when the population is at homeostasis, i.e. λ_0_ = *r*_2_(*p*_2_ − *q*_2_) = 0, we can show that Γ_1,2,1_ is larger than 1 and note that Γ_2,2,3_ is always positive, then Δλ_*S*_ is shown to be positive for any 0 ≤ *κ* ≤ 1. Fig 4 illustrates Δλ_*S*_ as a function of both *κ* and *p*_2_ in the scenario that Δλ_*S*_ can change from positive to negative. It is shown that with the increase of *p*_2_, the critical value κ* decreases, which means it is getting less likely for the *S* mutant cell population to be favored. Note that Γ_*j*,*k*,*l*_ represents the effect of cellular hierarchy on de-differentiation, and *κ* represents how de-differentiation reshapes the cellular division patterns. Therefore, the selection of de-differentiation is a combined result of cellular hierarchy and de-differentiation pattern.

We now turn our attention to the selection gradient (comparative fitness) of the *J* mutant cell population, which is given by (see S1 File for mathematical details)

$$\begin{array}{c} \Delta \lambda_{J}=\{\begin{array}{cc} (\prod_{i=1}^{j_{0}-1}\Gamma_{i,j_{0},i})(\prod_{i=j_{0}+1}^{n-1}\Gamma_{i-1,j_{0},i})\; & \text{for}\;1\leq j_{0}<n-1\\ \prod_{i=1}^{n-2}\Gamma_{i,n-1,i}-\kappa \; & \text{for}\;j_{0}=n-1 \end{array} \end{array}$$ (9)

**Fig 5:**
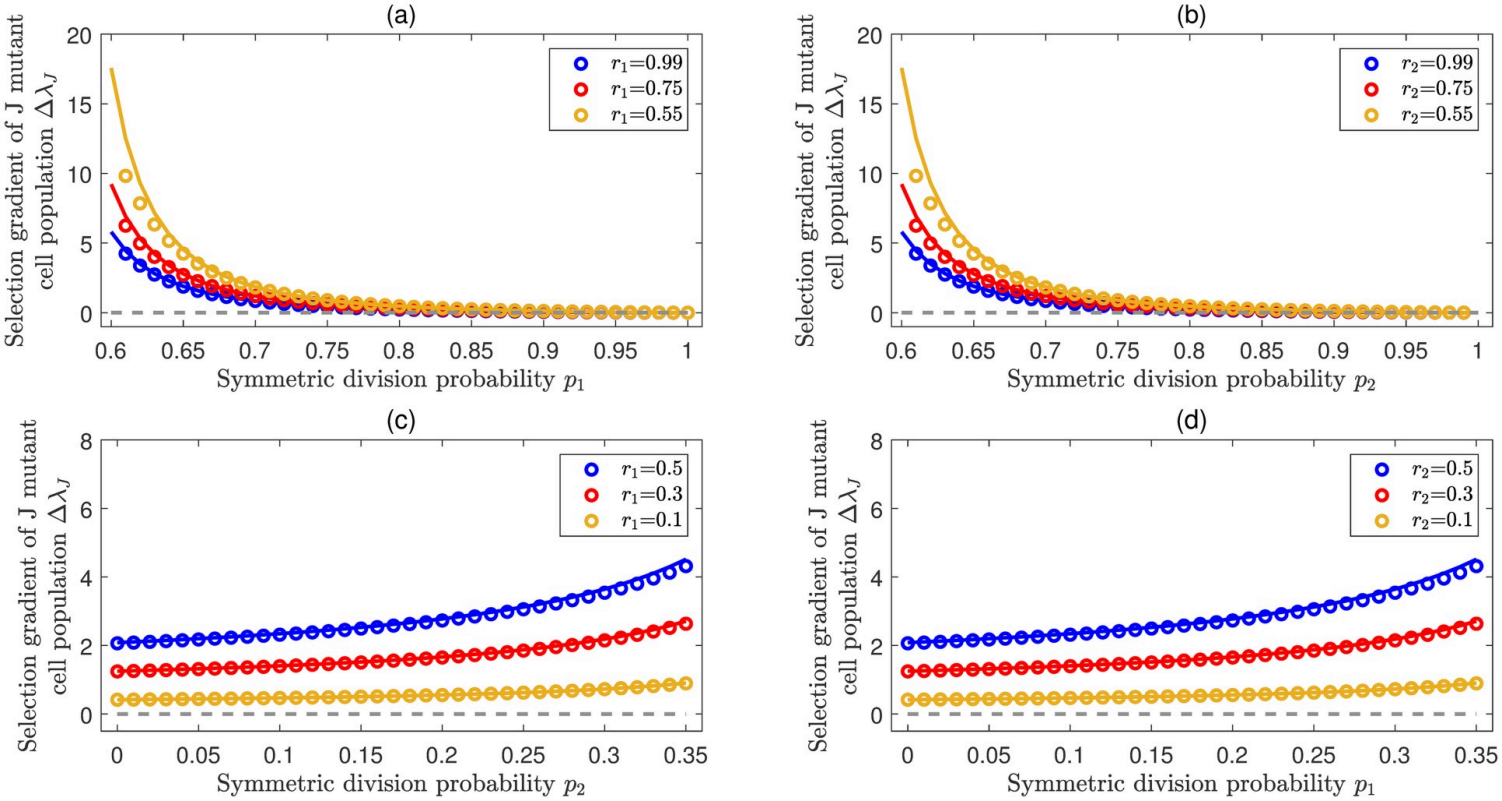
Selection for jumpwise de-differentiation. Illustrations of the selection gradient (comparative fitness) of the *J* mutant cell population Δλ_*J*_ for the cases *j*_0_ = 1 and *j*_0_ = 2. In all panels, colored lines represent analytical approximations from Eq (9) by using eigenvalue perturbation and symbols represent exact numerical solutions. The joint parameters *n* = 4, *κ* = 0.1, *ρ* = 0.01, *d* = 0.05. (**a**) Δλ_*J*_ as a function of *p*_1_ provided an expanding population in which compartment 1 has the largest effective self renewal rate, i.e. λ_0_ = *r*_1_(*p*_1_ − *q*_1_) > 0. Here *p*_2_ = 0.55, *p*_3_ = 0.6, *r*_1_ = 0.2, and *r*_3_ = 0.3. (**b**) Δλ_*J*_ as a function of *p*_2_ provided an expanding population in which compartment 2 has the largest effective self renewal rate, i.e. λ_0_ = *r*_2_(*p*_2_ − *q*_2_) > 0. Here, *p*_1_ = 0.55 *p*_3_ = 0.6, *r*_1_ = 0.2, *r*_3_ = 0.3. (**c**) Δλ_*J*_ as a function of *p*_2_ provided a steady population in which compartment 1 has the largest effective self renewal rate, i.e. λ_0_ = *r*_1_(*p*_1_ − *q*_1_) = 0. Here *p*_1_ = 0.5, *p*_3_ = 0.1, *r*_2_ = 0.4, and *r*_3_ = 0.6. (**d**) Δλ_*J*_ as a function of *p*_1_, provided a steady population in which compartment 2 has the largest effective self renewal rate, i.e. λ_0_ = *r*_2_(*p*_2_ − *q*_2_) = 0. Here, *p*_2_ = 0.5, *p*_3_ = 0.1, *r*_1_ = 0.4, and *r*_3_ = 0.6.

Similar to Eq (8), here all the Γ_*j*,*k*,*l*_ in Eq (9) are positive. For the case of 1 ≤ *j*_0_ < *n* − 1, in particular, Δλ_*J*_ is always positive, i.e. the *J* mutant cell population is advantageous. Fig 5 illustrates the selection of jumpwise de-differentiation for the cases *j*_0_ = 1 and *j*_0_ = 2. For each case, it is shown that Δλ_*J*_ is positive, regardless of whether the resident cell populations are expanding or maintaining homeostasis.

**Fig 6:**
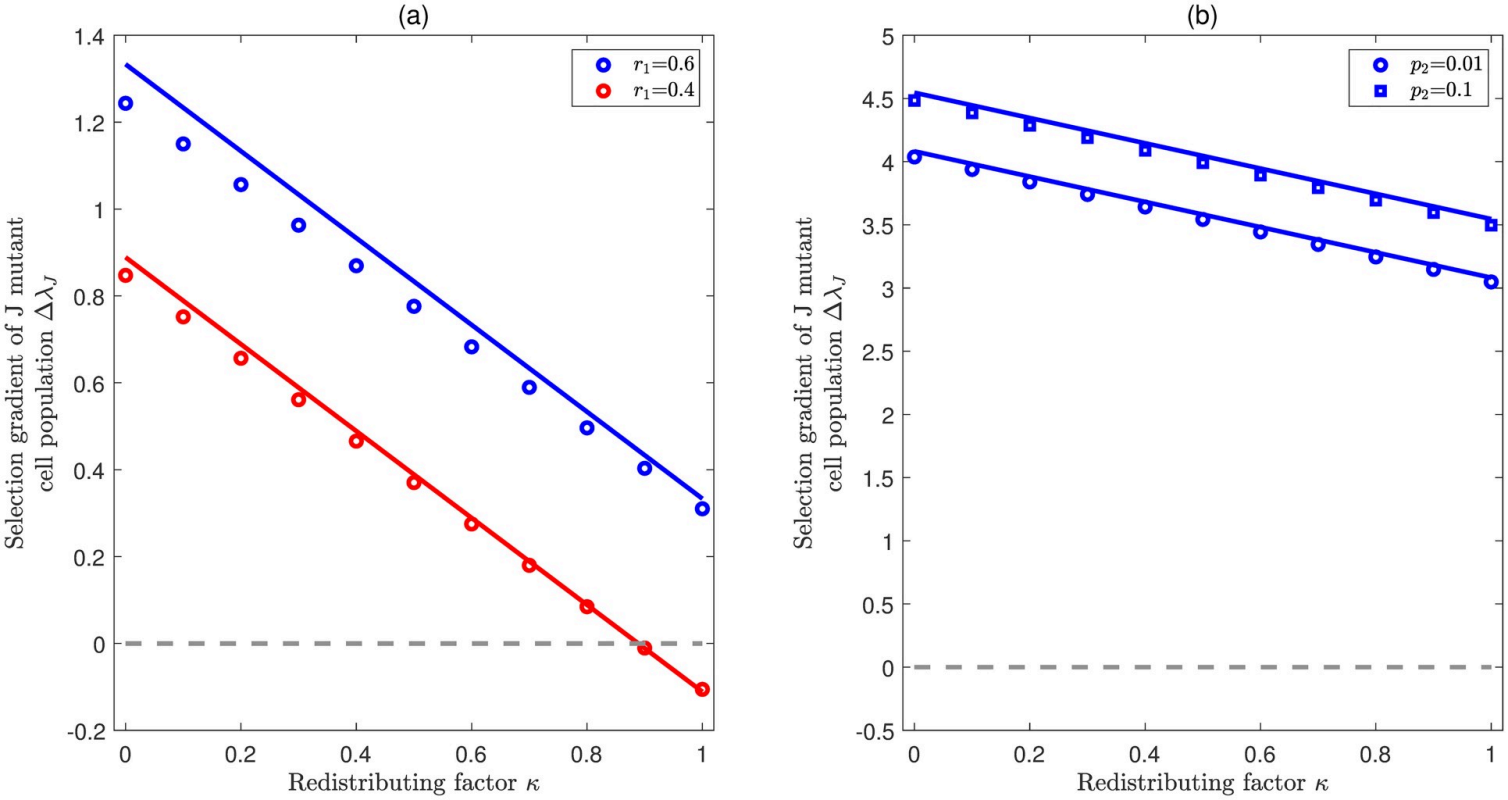
Selection for jumpwise de-differentiation when the effective rate of self renewal is highest in compartment 3. Illustration of the selection gradient Δλ_*J*_ as a function of the redistributing factor *κ* provided that λ_0_ = *r*_3_(*p*_3_ − *q*_3_). In both panels, colored lines represent eigenvalue perturbation results in Eq (9) and symbols represent exact numerical solutions. The common parameters are *n* = 4, *ρ* = 0.01, *d* = 0.05. (**a**) **Expanding case** (λ_0_ > 0). In this case, there are two different scenarios: For $r_{1}>\frac{r_{3}\left(2p_{3}-1\right)}{2\left(1-p_{1}\right)\Gamma_{2,3,2}+\left(2p_{1}-1\right)}\approx 0.45$, Δλ_*J*_ is always positive (blue color). For *r*_1_ < 0.45, Δλ_*S*_ is changed from positive to negative with the increase of *κ* (red color). Here *p*_1_ = 0.5, *p*_2_ = 0.65, *p*_3_ = 0.85, *r*_2_ = 0.4, *r*_3_ = 0.6. (**b**) **Homeostasis case** (λ_0_ = 0). In this case, Δλ_*J*_ is always positive. Here *p*_1_ = 0.01, *p*_3_ = 0.5, *r*_1_ = 0.8, *r*_2_ = 0.7, and *r*_3_ = 0.2.

On the other hand, for *j*_0_ = *n* − 1, Δλ_*J*_ is negatively correlated with the redistributing factor *κ*. Fig 6 illustrates how Δλ_*J*_ changes with *κ* provided that cells in compartment 3 have the largest effective self-renewal rate (*j*_0_ = *n* − 1 = 3). The results are quite similar to Fig 3. In the expanding case (left panel), either Δλ_*J*_ is always positive (blue line), or it changes from positive to negative at some critical point 0 < κ* < 1 (red line). Whereas in the homeostasis case (right panel), Δλ_*J*_ is always positive for all *κ* ∈ [0, 1]. Actually, when the largest eigenvalue becomes zero, theoretically we can show that Γ_*i*,*n* − 1,*i*_ is larger than 1, and then the product $\prod_{i=1}^{n-2}\Gamma_{i,n-1,i}$ is also larger than 1. In this way, $\Delta \lambda_{J}=\prod_{i=1}^{n-2}\Gamma_{i,n-1,i}-\kappa$ is always positive for any *κ* ∈ [0, 1]. By combining the results from Figs 3 and 6, we know that de-differentiation always provides a fitness advantage in the populations at homeostasis, regardless of how the redistributing factor *κ* affects the self-renewal and differentiation probabilities.

A comparison between Eqs (8) and (9) reveals some important differences between stepwise and jumpwise de-differentiation patterns. First of all, jumpwise de-differentiation provides a much wider range of favorable conditions for de-differentiation than stepwise de-differentiation in the sense that Δλ_*J*_ is always positive for any 1 ≤ *j*_0_ < *n* − 1, but Δλ_*S*_ is always positive only for *j*_0_ = 1. Secondly, Δλ_*S*_ only depends on the parameters related to the neighborhood compartments of *j*_0_, but Δλ_*J*_ depends on the parameters related to all compartments, ranging from the stem cell stage to the stage where de-differentiation occurs. This implies that, the total number of compartments does matter in the jumpwise case, but not in the stepwise case. In other words, stepwise de-differentiation utilizes the local structure around the compartment with the largest effective self-renewal rate, whereas jumpwise de-differentiation utilizes the global structure throughout the multi-compartment hierarchy.

## Discussion

In this study, we have explored the adaptive significance of de-differentiation in hierarchical multi-compartment structured cell populations. Favorable conditions for de-differentiation have been presented by comparing the fitness measures between resident hierarchical structured cell populations without de-differentiation and mutant cell populations with different modes of de-differentiation.

In principle, there are two main factors that could influence the selection of de-differentiation: cellular hierarchy and the de-differentiation pattern. Cellular hierarchy refers e.g. to the number of cell compartments, the inherent cell division pattern, and the cell division rate. These correspond to the parameter landscape of (*n*, *p*_*i*_, *q*_*i*_, *r*_*i*_) in our model. The de-differentiation pattern refers to different modes of de-differentiation (stepwise or jumpwise), as well as how de-differentiation reshapes the division pattern in the cellular hierarchy (corresponding to *κ* in our model). Interestingly, our results show that the selection gradients for de-differentiation (Δλ_*S*_ and Δλ_*J*_) can generally be decomposed into a sum of a cellular hierarchy part and a de-differentiation part, showing that the selection of de-differentiation is a result of the linear combinations of these two factors.

Among all factors in the cellular hierarchy, the most important one is which of the cell compartments has the largest effective self-renewal rate. In general the stem cells are the cells with the highest potential for long term self-renewal. There is also agreement that stem cells replicate slowly and therefore in many tissues it is the progenitor cells that lead to amplification and maintenance of tissues. There is evidence that cells downstream of the stem cells can undergo self-renewal, albeit not long term or indefinite. In hematopoiesis, for example, erythroid progenitors that are committed to produce red blood cells undergo self-renewal that is regulated by Bm1-1 and PU-1 [52, 53]. Guibal et al have also shown that proerythroblasts in the bone marrow undergo self-renewal [54]. Mutations in cells downstream of the hematopoietic stem cell can transform such cells with long term self-renewal potential behaving like stem cells and able to transfer disease in serial transplantation experiments. Examples of these include AML-ETO expression in primary erythroid cells [55], PML-RARA in acute promyelocytic leukemia [54]. Krivtsov et al [27] also discuss how MLL-AF7 expression in progenitor cells leads to stem cell like behavior. Finally, Jamieson et al have shown how the CML blast crisis emerges from progenitor cells not CML stem cells and leads to self-renewal of such transformed progenitor cells [56].

According to our results, de-differentiation is more likely to be favored when earlier compartments have the largest effective self-renewal rate. For example, in the stepwise case, de-differentiation is favored provided that stem cells have the largest effective self-renewal rate. This result is quite intuitive. Stem cells are normally considered to have the greatest self-renewal potential, and due to de-differentiation the stem cells compartment receives the influx from differentiated cells. In this way, de-differentiation contributes to a faster growth rate of the whole population. In the jumpwise case, de-differentiation is favored in all cases except when the latest divisible cell compartment has the largest effective self-renewal rate. Interestingly, these results apply in both expanding and steady cell populations. For the expanding case, advantageous de-differentiation can speed up the growth rate of the whole population. For the steady case, de-differentiating mutant cell populations with fitness advantage can escape from the homeostasis and expand with time. A significant biological implication of this result is that de-differentiation could play a very important role in tumor initiation [57] during which the balance between self-renewal and differentiation of stem cells could be broken. Furthermore, it has been reported that de-differentiation also happens in normal tissues and contributes to the regenerative processes after injuries [17, 19, 20]. Our results suggest that the presence of de-differentiation could effectively speed up the recovery of tissues. It should be noted that, even though the characteristics of de-differentiation seem similar in both tumorigenesis and regenerative processes, their biological mechanisms should be highly different: The de-differentiation in regenerative processes must be tightly regulated, whereas the de-differentiation in tumorigenesis may be more difficult to control. Note that the differences between them are still poorly understood, it will be very interesting and enlightening to model and compare de-differentiation mechanisms in these two different scenarios.

Given all the factors in the cellular hierarchy, we are most concerned about how different de-differentiation patterns shape the evolution of de-differentiation. In particular the redistributing factor, i.e. the effect of de-differentiation on self-renewal and differentiation probabilities greatly influences the selection conditions. Our results suggest that de-differentiation is more likely to be favored if there is less effect on self-renewal than on differentiation. That is, the smaller the redistributing factor *κ* is, the larger the selection gradient of de-differentiation will be. Furthermore, it should be noted that in the homeostasis cases, the selection gradients for both stepwise and jumpwise de-differentiation are always positive for any *κ* ∈ [0, 1], which suggests that de-differentiation is always advantageous when invading the hierarchical tissues at homeostasis. In addition, the de-differentiation mode (stepwise or jumpwise) has enormous implications for the selection conditions. Our results suggest that de-differentiation is more likely to be favored in the jumpwise case than in the stepwise case. However, jumpwise de-differentiation seems to be biologically much more difficult to achieve, the overall incidence of it would still be very low. Perhaps an example of the differences between stepwise and jumpwise de-differentiation and the implications of the subsequent disease behavior can be illustrated by various types of leukemia. As already mentioned, MLL-AF9 expression in committed progenitor cells can lead to the development of leukemic stem cells that can result in disease transmission across mice [27, 58]. In general MLL expression is associated with a poor prognosis in acute myeloid leukemia [59, 60]. This may be an example of jumpwise de-differentiation. In contrast, acute promyelocytic leukemia (APL) is an example of acute leukemia that is highly curable [61]. It is therefore possible that in this disease, stepwise de-differentiation—or a situation where a mutant cell can stick in a compartment without differentiating, similar to a stem cell—is occurring that in part makes the disease still potentially curable.

Note that the presented study is based on matrix population models with constant elements, which in principle do not take any non-linearity into account. Even though there are still uncertainties regarding the growth patterns of cell populations in different contexts (cancer or normal, solid or hematologic tumor, in vivo or in vitro) [7, 62] and linear models are often considered to be unable to capture the biological processes in reality, they are widely employed as default models to describe steady or growing cell populations, especially in normal tissue at homeostasis and early cancer development [21, 63–66]. We followed this idea and used it as a starting point to explore the adaptive significance of de-differentiation. In the future, more complex biological mechanisms such as non-linear feedback [67, 68] could be taken into account. In pioneering work, Wodarz studied mathematical models by integrating feedback regulation with de-differentiation [33]. He showed that in the presence of non-linear feedback, de-differentiation can lower the rates of tumor initiation and progression. Interestingly, this prediction is opposite to the prediction by Shirayeh et al [32], in which they showed that de-differentiation can increase the rate of tumor initiation in the absence of non-linear feedback. The discrepancy between these two predictions actually reveals the complexities brought by the non-linear feedback which deserves special attention in future study. Moreover, while the hierarchical architecture of tissues is considered to have been selected to minimize the risk of retention of mutations, the risk of acquisition of stem cell like properties by the large population of progenitor cells introduces new dynamics—perhaps in such a scenario two additional considerations could reduce the risk of cancer—namely the low probability that specific mutations lead to acquisition of stem cell like behavior or the average survival of progenitor cells may be low enough to prevent the acquisition of the additional mutations needed to reach the full cancer phenotype. This could be an extension of this work in future.

## Supporting information

S1 File Supplementary methods. (PDF)
